# A Possible Role of Anhedonia as Common Substrate for Depression and Anxiety

**DOI:** 10.1155/2016/1598130

**Published:** 2016-03-02

**Authors:** Luigi Grillo

**Affiliations:** Via Ragazzi del 99, No. 45, 20010 San Giorgio su Legnano, Italy

## Abstract

Depression and anxiety are often comorbid, in up to 70% of cases, and the level of one or the other may fluctuate, leading now to a diagnosis of depression, now to a diagnosis of anxiety. For these reasons, and for the presence of many other common factors, it has been suggested that both are part of the same continuum of problems and that they have a common substrate. This paper proposes the possibility that anhedonia may be an important component of this possible common substrate, and it tries to identify the mechanism with which anhedonia could contribute to causing both depression and anxiety. It also proposes an explanation why an intense pleasure could improve both depression and anxiety.

## 1. Introduction

Depression and anxiety are often comorbid, in up to 70% of cases [1], and the level of one or the other may fluctuate, leading now to a diagnosis of depression, now to a diagnosis of anxiety [2, 3]. Both are preceded and facilitated by stress, either recent [4–9] or dating back to childhood [10–12], they are often accompanied by the search for drugs [13–15], they are part of the symptoms of withdrawal from drugs [15], and they are associated with rumination (i.e., with lingering over the same gloomy thoughts) [16]. They are also characterized by anhedonia [17–22], a reduction of the ability to feel pleasures.

According to Cohen et al., “despite increased attention over the past 25 years on the comorbidity between depression and anxiety, there is still a great deal of uncertainty over why these two disorders are so highly comorbid” [23]. And according to Starr and Davila, “although several comorbidity theories exist, the most prominent ones attribute anxiety-depression comorbidity to shared structural components. For example, Clark and Watson's (1991) tripartite model hypothesizes that anxiety is uniquely defined by physiological hyperarousal and depression by anhedonia but that both disorders share the common thread of elevated negative affectivity. Other researchers have presented similar structural models, in which shared underlying factors account for depression-anxiety cooccurrence. The tripartite model and other structural models have greatly expanded our understanding of anxiety-depression comorbidity, providing a nuanced view of which aspects of anxiety and depressive symptoms are most likely to cooccur. However, these models do not sufficiently explain depression-anxiety cooccurrence, for several reasons. First, although a number of studies have supported the tripartite model's three factors, others have not. Second, studies have shown both that physiological hyperactivity is correlated with depression and that anhedonia is correlated with anxiety, contradicting the specificity hypothesis of the tripartite model and suggesting that cooccurrence is not entirely accounted for by the hypothesized shared substrates” [24].

Starr and Davila also propose a model of their own, in which “anxiety symptoms lead to depressive symptoms via maladaptive anxiety response styles,” bearing in mind that “many anxious people may develop hopeless thoughts about their anxiety” and that “in turn, several researchers have identified hopelessness and related attributions as a key risk factor for depressive symptoms” [24]. The “hopeless thoughts” of many anxious people seem to reveal that they live in a situation of continuous severe stress.

For their intermingling, and for the presence of many common factors, the possibility has been proposed that depression and anxiety are both part of the same continuum of problems [25, 26] and that they have a common substrate [27–29] that some authors suggest might be anhedonia [22].

The presence of anhedonia both in anxiety and in depression could be explained by the fact that both conditions are induced by stress [6, 30], and stress—acute or chronic—is regularly accompanied and followed by anhedonia in humans [9, 17, 31, 32] and in animals [33, 34].

The reduction of the ability to feel pleasure caused by stressful events tends to be long-lasting. For instance, traumatic experiences in infancy may cause anhedonia in adult life in man [35] and in animals [36]. Naturally, we are talking about significant anhedonia: low-grade anhedonia is normally present even in healthy people [37] who have not suffered severe stress, as everyday adversities are more or less stressful, and in some cases humiliation or feeling subordinate [38] or a low income [39] is enough to reduce a person's capacity for pleasure.

## 2. Anhedonia and Depression

Anhedonia is a key symptom of major depression [31, 40, 41] and it can influence many of its symptoms. Let us consider, for example, apathy in depressed people. Each action normally tends to provide pleasure [42]. Even freeing oneself from an unpleasant or painful situation is a pleasure [43], and this pleasure is at the basis of the attempts to escape from difficult situations [44, 45]. If we are unable to feel pleasures, as in the case of anhedonia, the desire to perform the actions necessary to achieve them is reduced or disappears [46] and may result in various levels of apathy [47].

Even difficulty in concentrating and keeping up attention, present in depression, could depend on anhedonia. In fact, the ability to feel pleasures seems to influence the ability to perceive and connect sensory information and the ability to learn. For example, in animals, when two separate stimuli, which individually do not provoke pleasure, are mentally connected to each other, the formation of this new bond causes pleasure, and this is considered important for learning in general [48, 49]. In man, satisfying a curiosity is pleasant [50], just as it is pleasant to grasp an inner meaning [51], and the pleasure connected to awaiting a novelty (novelties are pleasant [52]) facilitates learning [53]. So, at least in part, the reduced capacity to apply oneself encountered in depressed people could depend on the scarcity or lack of their ability to feel these pleasures. Moreover, a reduced hedonic tone is associated with a reduction of the cognitive processes both in the first automatic phase and in the subsequent phases of processing sensations [41].

Even rumination, which is frequent in depression [54], could derive from anhedonia: the ability to feel pleasures, in fact, allows an extension of both verbal [55–57] and visual [56, 57] associations and improves rumination [58]. Also the gloomy mood of depressed persons could be partially explained by the fact that they have to lead a life without everyday pleasures and they are unable to be active in a satisfactory way.

## 3. Pleasures and Depression

Various pleasant sensations can improve both anhedonia and depression, and this fact seems to be a further suggestion that anhedonia can influence many of the symptoms of depression. In man, drugs are used by anhedonic persons, with or without psychiatric disturbances, to try to reduce their reward deficit [59], and opioids have had an important role in the treatment of depression [60]. Depressed persons sometimes use sweet or fatty foods as self-medication, according to some authors to compensate the loss of the ability to feel normal pleasures [59, 61]. In rats, an enriched environment improves depression [62, 63] and so do sweet or fatty foods [64].

All these pleasures that are able to have a positive influence on anhedonia and depression have one particular feature: they give a pleasure more intense than ordinary everyday pleasures. The intensity of the pleasure produced by opioids and other drugs is well known, and also sweet or fatty foods are very strong pleasures for both men and animals [65–67]. Besides the possibility of physical exercise (which is an important pleasure if voluntary [68, 69]), an enriched environment also implies continuous novelties [63], and novelties provide strong pleasure [70, 71].

Since depression is characterized by anhedonia, perhaps these pleasures must be particularly intense to be able to overcome the high threshold for pleasure that characterizes anhedonia: in this way, a certain number of depressed people could feel them even when normal pleasures are no longer felt.

The mechanism with which a strong pleasure seems able to have a positive influence on depression is still not clear. An improvement of depression should mean regaining the ability to enjoy the ordinary pleasures of life, an ability that had been lost or very much reduced because of anhedonia. But how could a single pleasure, however intense it may be, for example, a dose of drugs, allow the recovery of sensitivity of many other different, less intense pleasures, much more normal and everyday, the loss of which is the central factor of depression?

An answer might be suggested by a particular aspect of the pleasant stimuli: besides giving pleasure, they also allow simultaneous or subsequent pleasures to be felt more intensely for a certain period. For example, a single dose of drugs causes an increase of sensitivity to subsequent pleasures, both to drugs [72, 73] and to normal pleasures such as food and social play [72, 74–78]. Normal pleasures in turn increase the pleasure given both by drugs [77, 79, 80] and by other normal pleasures [81], so that, in conclusion, “the perceived value of present reward is built upon past reward exposure” [81]. The high threshold for pleasure due to anhedonia prevents depressed persons from enjoying normal everyday pleasures, but some sufficiently intense pleasures could overcome this threshold, still be perceived, and be able to increase and recover other pleasures previously faded or lost. In this way, anhedonic persons could recover the possibility of enjoying ordinary everyday pleasures and leading a more pleasant life for some time. This could explain the fact why only particularly intense pleasures seem to be able to improve anhedonia and depression.

## 4. Stress, Dynorphin, and Anhedonia

Many of the pleasures that are able to improve depression also improve anxiety. For example, in man, opioids can relieve both depression and anxiety [60, 82], and alcohol is sought to cope with both conditions [83–89]. In animals, an enriched environment [63, 90–93] and agreeable foods [94–96] can improve depression and anxiety. This could suggest that anhedonia might contribute to causing, in addition to depression, even anxiety. But if it is fairly understandable that anhedonia could cause many of the symptoms of depression [97], it is difficult to imagine how anhedonia could cause anxiety.

At this point, it could be useful to recall the mechanism with which stress can provoke anhedonia. Some hormones are released during intense or repeated stress: adrenalin and noradrenaline which help to combat stress by increasing the flow of blood and glucose to the muscles and the brain, and corticotropin-releasing hormone (CRH) which in turn provokes the release of cortisone, the action of which accompanies and reinforces that of adrenalin and noradrenaline. The CRH also releases another hormone, dynorphin [97–99], which reduces the release of dopamine in the nucleus accumbens [100, 101]. The dopamine released in the nucleus accumbens produces pleasure [44, 102], so that stress can reduce the ability to perceive pleasure by reducing dopamine through the action of dynorphin. (As well as dopamine, opioids too are involved in feeling pleasure [103], and it has been proposed that dopamine is more involved in the search for pleasure, while opioids are involved in its enjoyment [104]. However, the action of dopamine and that of opioids are closely interwoven and overlapped, since dopamine releases endogenous opioids [105–108], while the opioids in turn release dopamine in the nucleus accumbens [105]. In this chain of interconnections between dopamine and endogenous opioids, some authors believe that dopamine could be the “basic link” [109].) On account of its ability to cause anhedonia, dynorphin is considered to be responsible for depression due to stress [14].

Dynorphin is released not only in the case of stress, but also in the case of an excessive release of dopamine, for example, when taking drugs. All drugs cause a marked increase of dopamine in the nucleus accumbens [100, 102, 107], and the intense pleasure that they procure is in most cases the reason why they are initially sought [110, 111]. However, if the use of drugs continues, the pleasure is reduced more and more. This reduction seems to be a consequence of the excessive pleasure of the drugs: to counterbalance the excessive dopamine stimulus, which can be harmful, dynorphin is released [100, 112, 113], which reduces the dopamine with consequent anhedonia. The effect of dynorphin is long-lasting [100, 113], and it is considered responsible, not only for anhedonia, but also for withdrawal symptoms (anxiety, agitation, depression, etc.) which are perceived when the effect of the drug ceases [113]. To rid oneself of withdrawal symptoms it is necessary to take the drug again, even though the pleasure that it procures is very much reduced because of anhedonia. At this point the search for drugs becomes more and more compulsive and, from an optional way of behaviour, aimed at obtaining pleasure, it is transformed into a forced behaviour aimed at ridding the user of the painful states of distress and suffering that accompany withdrawal [110, 114, 115]. This is how addiction begins [116].

Among the negative states that are reported as hard to bear in the withdrawal syndrome, anxiety is particularly stressed and is considered a consequence of dynorphin. In fact, dynorphin provokes both anhedonia and anxiety. Its anxiogenic effect has not been considered in relation to anhedonia but has been considered a consequence of a direct action of dynorphin on the anxiogenic centres (in particular on the amygdala, through dynorphinic receptors—KOR, *κ*-opioid receptors—present in the amygdala) [97].

However, this possible direct action of dynorphin does not explain the fact that many substances or situations that provoke intense pleasures possess an anxiolytic effect: as we have seen, opioids and alcohol can improve anxiety in man, while in animals pleasant foods, an enriched environment, and alcohol have the same positive effect on anxiety. It is perhaps worth considering the possibility that a possible direct action of dynorphin on the anxiogenic centres may be accompanied by an indirect anxiogenic action mediated by the anhedonic effect of dynorphin. Some authors leave this possibility open: “Given the high comorbidity of depressive and anxiety disorders, KOR signalling and control of DA function may underlie the pathogenesis of both” [6]. A possible anxiogenic action through a reduction of DA (dopamine) function and consequent anhedonia would allow the unification of the action mechanism of dynorphin on both anxiety and depression and could help explain why an intense pleasure is able to improve both depression and anxiety. But how could anhedonia arouse anxiety, that is, arouse an inexplicable fear of a grave imminent and unknown danger?

## 5. Anhedonia and Anxiety

The image of the world we receive through what we see and hear has to be continuously reassembled and reorganized; otherwise it could become distorted and fragmented and could change so much that we hardly recognize it. As regards the visual aspect, “our perceptual impressions of an object and its context are in permanent flux as we move or as the object moves or transforms itself: the (perceived) world is not static but permanently physically changing” [117]. We must immediately realize that a foreshortening is not a real deformation, that an object that is partially hidden is not cut off but extends under the cover, that a cloud seen through the branches of a tree is not part of the tree, and so on. For example, every time we look at a three-dimensional object from a different angle “the observer should be expected to see an object of changing shape. The cube should undergo constant amoebic transformations… Fortunately, but surprisingly enough, this does not happen” [118, p. 71]. But perhaps this might happen if something interferes with the mechanism of the indispensable continuous reorganization and interpretation of an “amoebic” reality. In this case, the alterations might become evident, objects might seem deformed and frightening, sky and clouds might seem head-spinningly close to the observer, making the surrounding world flat and oppressive, and so on.

One of the possible factors that might interfere with the indispensable and continuous reconstruction of the world could be anhedonia. We have seen how, according to many researchers, our ability to feel pleasure can influence our ability to perceive and connect sensory information. As regards the visual aspect in particular, recognizing an expected image gives pleasure, and if the image is unexpected the pleasure is even greater [119]. Recognizing a shape in an ambiguous context gives pleasure [51] and this stimulates us to look for other ambiguous shapes to discover their correct shape [117]. Getting rid of visual ambiguity gives pleasure in the same way as escaping a danger, on condition that we feel sufficiently protected in the moments of uncertainty that accompany the attempt to understand [120]. Visual learning is also facilitated by a simultaneous pleasure, even if this pleasure is completely extraneous to vision [121].

If we do not feel these pleasures, as may happen in some cases of marked anhedonia, our ability continuously to correct and interpret the variable aspect of our surroundings might be reduced and various degrees of frightening alteration of the aspect of the environment might result. Even in a person without psychiatric pathologies, the sudden awareness of a deformation of the appearance of the environment, deformation that had always been present but had previously been ignored, may give rise to intense anxiety. An example of this can be found in “art and visual perception,” a book by Rudolf Arnheim, former president of the Division on Psychology and the Arts of the American Psychological Association, where the author describes the distressed reaction of a student when, following a suggestion by the teacher, she became aware of the deformations assumed by the appearance of an object according to the viewpoint from which it was observed, deformations which she had always corrected automatically without realizing it. “It is very difficult for many persons to visualize the working of perspective, even when it is demonstrated to them with a yardstick. Recently an intelligent and sensitive young college student, to whom I tried to show the oblique shape of a box on the table, finally hid her face in sudden terror and exclaimed: ‘It is true - how horrible!'” [118, p. 160]. And in the case reported by Arnheim it was only a completely explainable alteration of the appearance of a single small object.

Perhaps even just a very brief, but unexpected and inexplicable deformation of some aspect of the environment caused by anhedonia might give in some cases the sensation that the apparent normality of the objects is only a fragile veil, which could be torn and reveal inexplicable and frightening deformities. Even only the vague feeling that this might happen might induce indefinable, inexpressible fear of imminent catastrophe, generating a state of anxiety, the cause of which cannot be explained to other people because the sufferers cannot explain it to themselves.

In any case, the hypothesis that a reduction of the pleasures connected with vision, or with other sensory information, could create difficulties in the indispensable, unceasing reconstruction of an environment that is continuously “physically changing” [117], and that this could contribute to the onset of anxiety might perhaps help us explain why an intense pleasure is able to relieve temporarily not only depression but also anxiety.

## 6. How a Pleasure, Sufficiently Intense to Be Felt, Could Improve Not Only Depression but Also Anxiety?

If we accept that anhedonia can contribute, through sensory alterations and consequent alterations of the environment, to the onset of the anxiety that accompanies depression, it becomes possible to propose an explanation of the reason why some intense pleasures improve anxiety as well as depression: their antianxiety action could depend, like the antidepressive action, on their ability to make lost or faded pleasures be perceived again. In fact, if the recovered pleasures also included those felt when perceiving and correctly connecting sensory information, these pleasures might help to recover the normal, indispensable ability to reconstruct the continuously variable aspect of the environment, which would thus be safe and familiar again. In this way, a sufficiently intense pleasure could reduce, with anhedonia, both depression and anxiety and help to lead a normal life again, at least for a certain time.
